# Homologous booster immunization with an inactivated vaccine induced robust antibody response in healthcare workers: A retrospective study

**DOI:** 10.3389/fimmu.2023.1099629

**Published:** 2023-02-03

**Authors:** Gui-Ping Wen, Min Zhu, Li-Rong Li, Xiu-Juan Li, Hui-Ming Ye, Yu-Lin Zhou

**Affiliations:** ^1^ United Diagnostic and Research Center for Clinical Genetics, Women and Children’s Hospital, School of Medicine and School of Public Health, Xiamen University, Xiamen, Fujian, China; ^2^ Department of Clinical Laboratory, Women and Children’s Hospital, School of Medicine, Xiamen University, Xiamen, Fujian, China; ^3^ Department of Hospital Infection Management, Women and Children’s Hospital, School of Medicine, Xiamen University, Xiamen, Fujian, China

**Keywords:** SARS-CoV-2, inactivated vaccine, booster vaccination, immune persistence, antibody response

## Abstract

Coronavirus Disease 2019 (Covid-19) severely impacted the health, society, and economy around the world. With declining protective efficacy of primary vaccination and the spread of severe acute respiratory syndrome coronavirus 2 (SARS-CoV-2) variants, a Covid-19 booster vaccination is being fully implemented globally. Many people received three doses of BBIBP-CorV inactivated vaccine in China and other developing countries. However, the antibody response and immune persistence of the homologous BBIBP-CorV booster vaccination is yet to be thoroughly evaluated, as previous studies focused within one month after the third dose. In this study, 97 participants were enrolled to analyze the antibody response and immune persistence within 6 months as well as the safety within 7 days after the third-dose of homologous BBIBP-CorV inactivated vaccine. The seroconversion rate for total antibody against the receptor binding domain (RBD) of the SARS-CoV-2 spike (S) protein were both 100% at month 1 and month 6 after the third dose. The IgG against the RBD of the SARS-CoV-2 S protein seroconversion rate increased from 42.27% before the third dose to 100% 1 month after the third dose and then slightly decreased to 98.97% 5 months later. Positive IgM against the RBD of the SARS-CoV-2 S protein was rare and was observed in only one participant at month 1 after the third dose. The neutralizing antibody levels at month 1 and month 6 after the third dose increased 63.32-fold and 13.16-fold compared with those before the third dose, and the positive rate for neutralizing antibody was still 100% at month 6 after the third dose. Importantly, the antibody responses induced by the vaccine and immune persistence were not affected by sex or age. No serious adverse reactions were reported. Total antibody and IgG against the RBD of the SARS-CoV-2 S protein were highly correlated with neutralizing antibody, suggesting that total antibody and IgG against the RBD of the SARS-CoV-2 S protein could be used as predictors for neutralizing antibody. In conclusion, the third dose of homologous BBIBP-CorV inactivated vaccine induced a robust antibody response and moderate immune persistence. These finding are of great significance for development future vaccination strategies.

## 1 Introduction

The ongoing coronavirus disease 2019 (Covid-19) pandemic caused by severe acute respiratory syndrome coronavirus 2 (SARS-CoV-2) has caused serious damage to global public health and the economy (1). Although the disease is mild in most cases, it progresses to a severe form in some patients and may lead to deaths, especially in the elderly and people with comorbidities. According to the World Health Organization (WHO), as of October 3, 2022, SARS-CoV-2 resulted in more than 615 million laboratory-confirmed cases and over 6.5 million deaths (2).

Vaccines are considered as an economical and effective means for prevention and control of SARS-CoV-2. The vaccines against SARS-CoV-2 have been proven to be safe and reduce symptomatic infections and asymptomatic infections of SARS-CoV-2 as well as the adverse outcomes (3–7). It’s worth noting that vaccine-induced antibody titers and protective efficacy of Covid-19 vaccines declined over time, regardless of vaccine type (6, 8, 9). Additionally, variants of SARS-CoV-2 have spread globally, causing resurgences of infections even in countries and areas with successful mass-vaccination campaigns (6). Due to the decline of vaccine efficacy coinciding with the rapid spread of SARS-CoV-2 variants, a booster vaccination has been implemented in many countries, in which a third dose of Covid-19 vaccine was administered in people who had received a second dose.

Inactivated SARS-CoV-2 virus vaccines have been widely applied in China and many other countries. As an inactivated vaccine, the BBIBP-CorV vaccine has shown a vaccine efficacy of 78.1% in adults in the phase III clinical trial with a two-dose schedule and was proven to be safe and well tolerated in people aged 3-17 years, people aged 18-59, people aged ≥60, and individuals with comorbidities (4, 10–12). The BBIBP-CorV vaccine has been approved for conditional use in China and is included in the WHO emergency use listing (13) (14). Given that the vaccination was cost-effective in low- and middle-income countries (15), a vaccination strategy with BBIBP-CorV inactivated vaccine would bring important economic benefits in these countries. A third dose of homologous BBIBP-CorV vaccine showed a satisfying safety profile and induct robust humoral responses against SARS-CoV-2 infection (10, 16–20). However, the humoral response and immune persistence of a third dose of homologous BBIBP-CorV vaccine have not been fully explored, as previous studies focused within 1 month after the administration of the third dose (10, 16–20). The 6-month durability of the humoral immune response in vaccine recipients was still unknown and immune persistence was still under investigation.

In this study, 97 participants who had no previous SARS-CoV-2 infection and received three doses of BBIBP-CorV inactivated vaccine were enrolled. The antibody response and immune persistence within 6 months as well as the safety within 7 days in these participants were detailed assessed.

## 2 Materials and methods

### 2.1 Study design and participants

The healthcare workers were enrolled from the Women and Children’s Hospital, School of Medicine, Xiamen University. Theses participants received total 3 doses (0.5 mL per dose) of BBIBP-CorV inactivated vaccine (Beijing Institute of Biological Products Co., Beijing, China). Each dose of the BBIBP-CorV inactivated vaccine contained 4 μg of total proteins with 0.45 mg/mL aluminum hydroxide adjuvant. Two doses were given with an interval of 4 weeks. Then, a third dose was given 7-10 months after the second dose. The serum samples were collected from the participants at three time points, before the third dose and 1 month and 6 months after the third dose, between October 2021 and May 2022. A total of 97 participants provided serum samples at three time points and these 97 participants were enrolled and retrospectively analyzed for the antibodies against SARS-CoV-2. This study was a retrospective cohort study. The study was done in accordance with the Declaration of Helsinki and was reviewed and approved by the Medical Ethics Committee of the Women and Children’s Hospital, School of Medicine, Xiamen University. Written informed consent was obtained from all participants.

### 2.2 Antibody measurement

The receptor binding domain (RBD) of the SARS-CoV-2 spike (S) protein was the major target for neutralizing antibody and an attractive vaccine target because of its immunodominance (21). Therefore, the antibody response to the RBD of the SARS-CoV-2 S protein were analyzed in this study. The total antibody, IgM antibody, and IgG antibody against the RBD of the SARS-CoV-2 S protein in serum samples were tested using commercial chemiluminescence microparticle immunoassay kits (Xiamen InnoDx Biotech Co., Xiamen, China) according to the manufacturer’s instructions. The measurement processes were conducted with an automatic analyzer Caris 200 (Xiamen UMIC Medical Instrument Co. Ltd., Xiamen, China). A double-antigen sandwich immunoassay was used to detect total antibody and a μ-chain capture immunoassay was used to detect IgM antibody as previously reported (22). The total antibody and IgM antibody assays were established using recombinant antigens containing the RBD of the SARS-CoV-2 S protein (22). The IgG antibody kits were indirect immunoassays with a recombinant RBD of the SARS-CoV-2 S protein as the coating antigen. The amino acids reference sequences of the RBD and S protein of SARS-CoV-2 were from Wuhan-Hu-1 strain (prototype) (GenBank: NC_045512). The chemiluminescence reaction was measured by the Caris 200 as relative light units (RLUs). The RLUs were proportional to the content of total antibody, IgM antibody, and IgG antibody against the RBD of the SARS-CoV-2 S protein in the sample. The cut-off values of total antibody, IgM antibody, and IgG antibody were determined according to the manufacturer’s instructions. The cut-off values of total antibody, IgM antibody, and IgG antibody were determined by RLUs of negative control plus (RLUs of positive control multiplied by 0.3), RLUs of negative control plus (RLUs of positive control multiplied by 0.2), and RLUs of negative control plus (RLUs of positive control multiplied by 0.5), respectively. The results of total antibody, IgG, and IgM were records as signal to cut-off (S/CO) and the values of S/CO≥1.0 were considered as positive.

The neutralizing antibody against SARS-CoV-2 in serum samples was also tested using a commercial chemiluminescence microparticle immunoassay kit (Xiamen InnoDx Biotech Co., Xiamen, China) according to the manufacturer’s instructions. The neutralizing antibody assays were performed on the automatic analyzer Caris 200. The neutralizing antibody assays were based on a competitive method. Neutralizing antibody against SARS-CoV-2 in the sample bind to the acridinium ester conjugated SARS-CoV-2 S protein. The acridinium ester conjugated SARS-CoV-2 S protein not neutralized by the SARS-CoV-2 neutralizing antibody forms a complex with biotinylated SARS-CoV-2 specific antibody, which binds to the streptavidin coated on the microparticle. The RLUs were inversely proportional to the content of SARS-CoV-2 neutralizing antibody in the sample. The neutralizing antibody levels were calibrated and traceable to the first WHO International Standard for anti-SARS-CoV-2 immunoglobulin (NIBSC code 20/136) and the results were shown as international units Per milliliter (IU/mL). The cut-off value was 11.50 IU/mL according to the manufacturer’s instructions.

### 2.3 Safety

Injection site adverse reactions and systemic adverse reactions in participants within 7 days after the third dose of BBIBP-CorV vaccine were collected using an electronic questionnaire. The questionnaire was administered by participants. The adverse events were graded according to the guiding principles for grading standard of adverse events in clinical trials of vaccines issued by NMPA (23).

### 2.4 Statistical analysis

Statistical analyses were carried out using IBM SPSS statistics (SPSS, IL, USA) and GraphPad Prism version 8.00 (GraphPad Software, CA, USA). Categorical data were summarized as counts and percentages. Continuous data were reported as mean ± standard deviation (SD) or median with interquartile range (IQR). The differences were calculated by the Wilcoxon matched-pairs sighed rank test, Kruskal-Wallis test, or Mann-Whitney U test. Spearman correlation analysis was used to determine the relationship among total antibody and IgG antibody against the RBD of SARS-CoV-2 S protein and neutralizing antibody. All serum samples collected at three time points were included the Spearman correlation analysis, respectively. P values were calculated by a two-tailed test and P values of 0.05 or lower were considered statistically significant.

## 3 Results

### 3.1 Characteristics of enrolled participants

**Figure 1:**
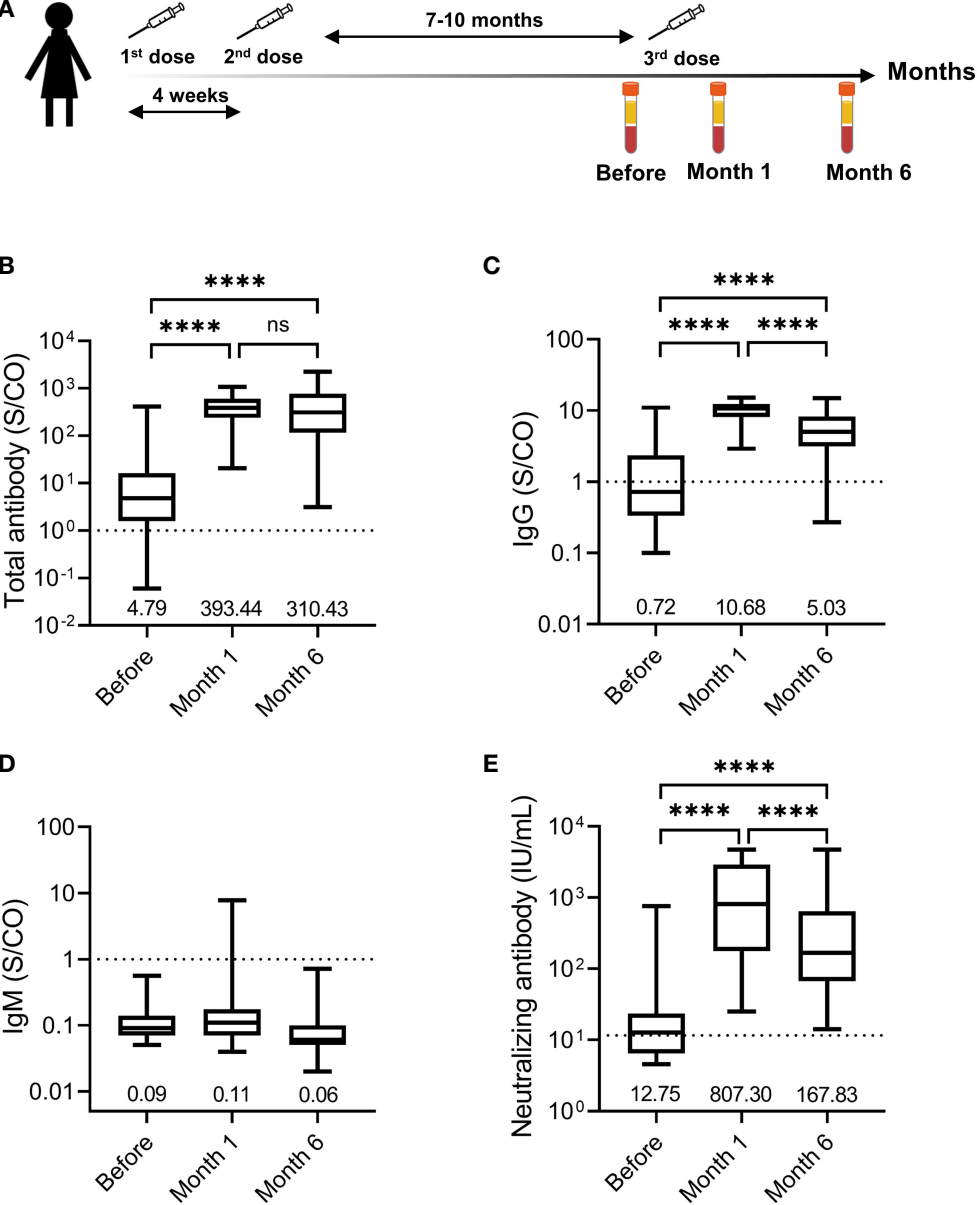
Humoral response induced by the third dose of homologous BBIBP-CorV inactivated vaccine. **(A)** Immunization regimen and time points of samples collected. The first, second, and third doses are indicated above the time axis with needles, respectively. Three time points selected for serum samples collection are indicated below the time axis with blood collection tube. Levels of total antibody **(B)**, IgG antibody **(C)**, and IgM antibody **(D)** against the receptor binding domain (RBD) of the SARS-CoV-2 spike (S) protein and neutralizing antibody **(E)** against SARS-CoV-2 are shown as the range (whiskers), interquartile range (boxes) and median (line within the boxes) values. The median levels of total antibody, IgG antibody and IgM antibody against the RBD of the SARS-CoV-2 S protein and neutralizing antibody against SARS-CoV-2 are shown at the bottom of each panel. The dashed horizontal lines indicate the cut-off of total antibody, IgG antibody and IgM antibody against the RBD of the SARS-CoV-2 S protein and neutralizing antibody against SARS-CoV-2. Comparisons were done by Wilcoxon matched-pairs sighed rank test **(B-E)**. ****p < 0.0001; ns, not significant.

Ninety-seven individuals received three doses of homologous BBIBP-CoV inactivated vaccine and provided blood samples at three time points before and after the third dose (**Table 1** and **Figure 1A**). These 97 participants were included in this study. The age of the participants ranged from 23 years to 57 years, with a mean age of 40.66±7.98 years, and 24 (24.74%) were males. Among the 97 participants, 100% were of Han ethnicity. The mean body-mass index (BMI) of the participants was 22.69±4.28 kg/m^2^. These participants had no underlying diseases, such as hypertension, cancer, or immune diseases.

**Table 1:** Baseline characteristics and antibodies against SARS-CoV-2 in the participants.

Characteristics	Participants (n=97)
Age (years), mean±SD	40.66±7.98
Sex (Male:Female)	24:73
Han ethnicity, n (%)	97 (100.00)
Body-mass index (kg/m^2^), mean±SD	22.69±4.28
Interval between 2^nd^ and 3^rd^ doses (days), mean±SD	257.34±8.75
Before the third dose	
Positive for total antibody, n (%)	81 (83.51)
Positive for IgM, n (%)	0 (0.00)
Positive for IgG, n (%)	41 (42.27)
Positive for neutralizing antibody, n (%)	54 (55.67)
Month 1 after the third dose	
Positive for total antibody, n (%)	97 (100.00)
Positive for IgM, n (%)	1 (1.03)
Positive for IgG, n (%)	97 (100.00)
Positive for neutralizing antibody, n (%)	97 (100.00)
Month 6 after the third dose	
Positive for total antibody, n (%)	97 (100.00)
Positive for IgM, n (%)	0 (0.00)
Positive for IgG, n (%)	96 (98.97)
Positive for neutralizing antibody, n (%)	97 (100.00)

### 3.2 Antibody response after the third dose

Overall, the third dose of homologous BBIBP-CoV inactivated vaccine induced a vigorous anti-SARS-Cov-2 response in most participants. The positive seroconversion of the total antibody was 83.51% (81/97) before the third dose and maintained at 100.00% (97/97) at month 1 and month 6 after the third dose (**Table 1**). The levels of the total antibody against the RBD of the SARS-CoV-2 S protein increased from 4.79 (1.57-16.40) S/CO before the third dose to 393.44 (238.76-608.58) S/CO at month 1 after the third dose and then declined to 310.43 (114.84-770.63) S/CO 5 months later (**Figure 1B**). For the IgG against the RBD of the SARS-CoV-2 S protein, the positive conversion rate increased from 42.27% before the third dose to 100.00% at month 1 after the third dose and slightly decreased to 98.97% at month 6 after the third dose. The levels of IgG against the RBD of the SARS-CoV-2 S protein increased from 0.72 (0.34-2.35) S/CO before the third dose to 10.68 (8.12-12.41) S/CO at month 1 after the third dose and decreased by approximately 50% at month 6 after the third dose (**Figure 1C**). The positive IgM against the RBD of the SARS-CoV-2 S protein was observed in only one (1.03%) participant at month 1 after the third dose (**Figure 1D**), suggesting that IgM response induced by the third dose of homologous BBIBP-CoV vaccine was rare.

We further analyzed the neutralizing antibody response in the participants. The seropositive rate of neutralizing antibody was 55.67% (54/97) before the third dose. The seroconversion of neutralizing antibody was observed in 100% (97/97) of the participants both at month 1 after the third dose and at month 6 after the third dose. The median neutralizing antibody levels increased 63.32-fold, from a base value of 12.75 (6.49-23.53) IU/mL before the third dose to 807.30 (177.08-2918.93) IU/mL at month 1 after the third dose (**Figure 1E**). Five months later, the median neutralizing antibody levels decreased to 167.83 (66.94-642.55) IU/mL, which was still 13.16-fold higher than that before the third dose. These results demonstrated that the neutralizing antibody induced by a third-dose homologous BBIBP-CorV booster vaccination could persist for at least 6 months in all participants.

### 3.3 Relationship between antibody response and age and sex

**Figure 2:**
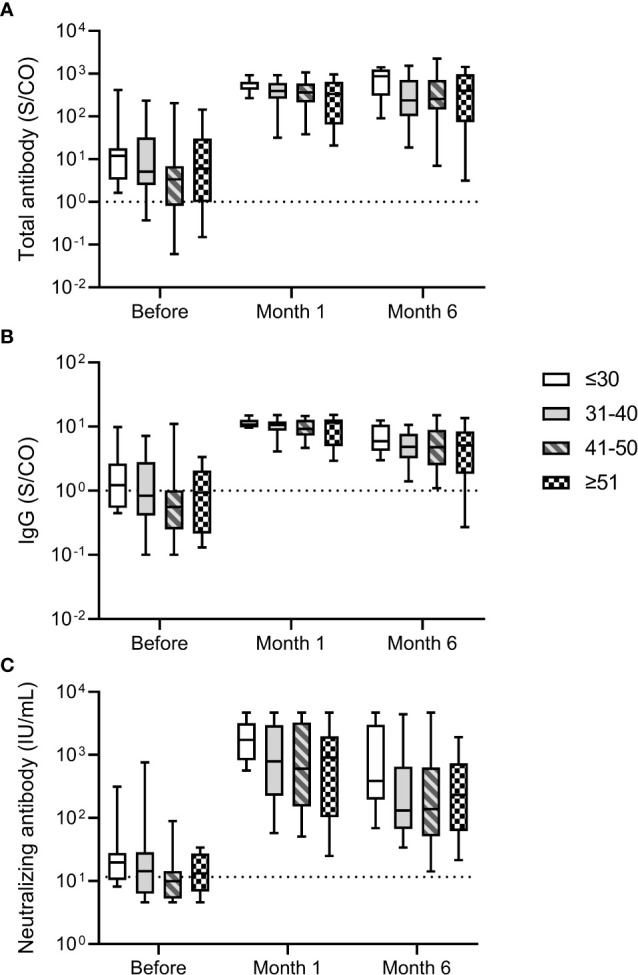
Antibody response induced by the third dose of homologous BBIBP-CorV inactivated vaccine in participants of different ages. The levels of total antibody **(A)** and IgG antibody **(B)** against the receptor binding domain (RBD) of the SARS-CoV-2 spike (S) protein and neutralizing antibody **(C)** against SARS-CoV-2 after the third dose are shown as the range (whiskers), interquartile range (boxes) and median (line within the boxes) values. The dashed lines represent the cut-off for total antibody and IgG antibody against the RBD of the SARS-CoV-2 S protein and neutralizing antibody against SARS-CoV-2. Comparisons were done by Kruskal-Wallis test.

We analyzed the relationship between antibody response and age of participants. No significant differences in total antibody and IgG against the RBD of SARS-CoV-2 S protein and neutralizing antibody levels were noted for participants of different ages before and after the third dose (**Figure 2**). The median neutralizing antibody levels at month 6 after the third dose were 387.24 (195.86-3007.66), 131.03 (66.84-653.31), 138.16 (51.19-632.73), 232.63 (61.75-739.55) IU/mL for participants aged ≤30, 31-40, 41-50, and ≥51 years, respectively. These results suggested that the antibody response induced by the third dose of homologous BBIBP-CoV vaccine was not affected by the age.

**Figure 3:**
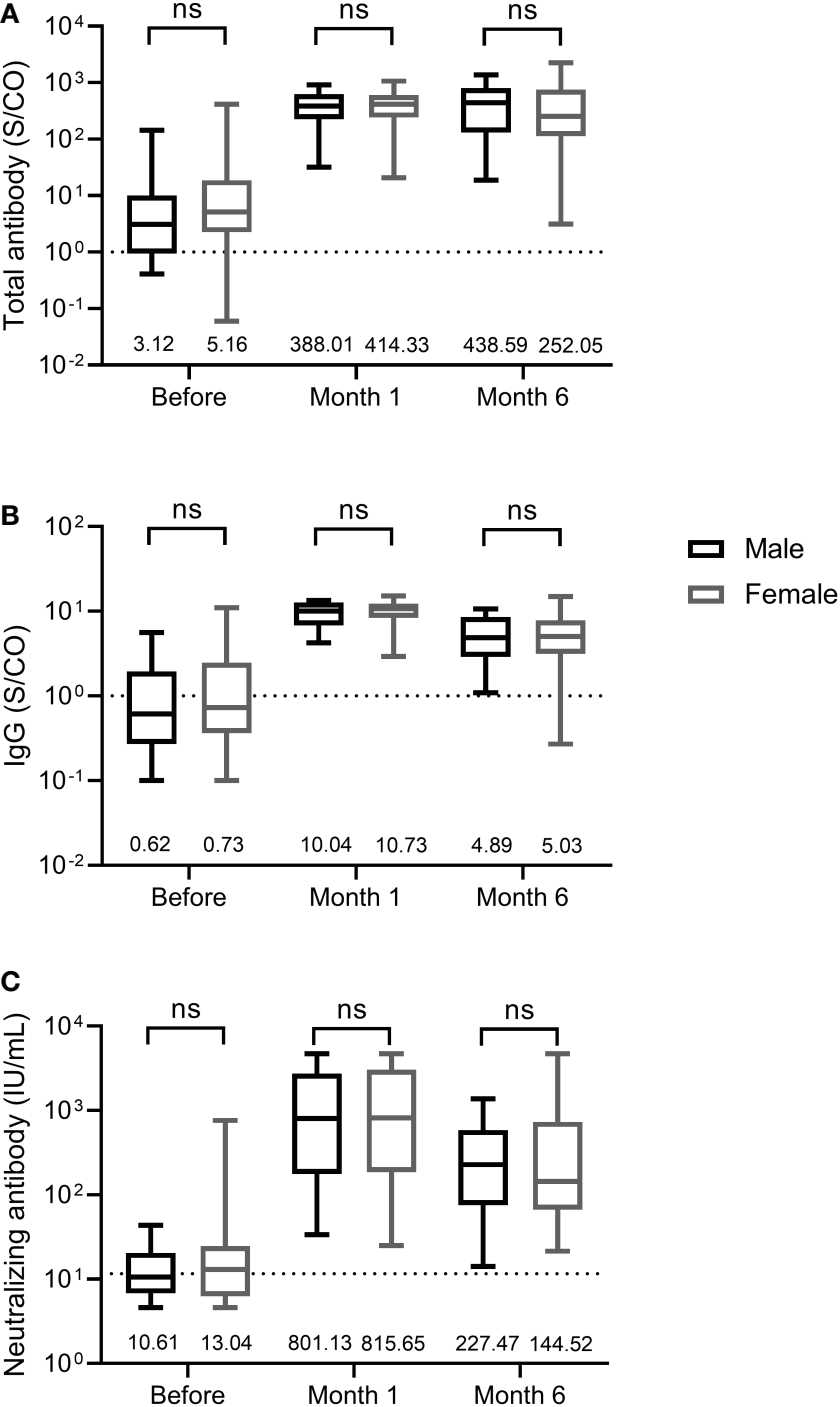
Antibody response induced by the third dose of homologous BBIBP-CorV inactivated vaccine in female and male participants. The levels of total antibody **(A)** and IgG antibody **(B)** against the receptor binding domain (RBD) of the SARS-CoV-2 spike (S) protein and neutralizing antibody **(C)** against SARS-CoV-2 are shown as the range (whiskers), interquartile range (boxes) and median (line within the boxes) values. The median levels of total antibody and IgG antibody against the RBD of the SARS-CoV-2 S protein and neutralizing antibody against SARS-CoV-2 are shown at the bottom of each panel. The dashed lines indicate the cut-off of total antibody and IgG antibody against the RBD of the SARS-CoV-2 S protein and neutralizing antibody against SARS-CoV-2. Comparisons were done by Mann-Whitney U test **(A-C)**. ns, not significant.

We further compared the differences in total antibody, IgG, and neutralizing antibody levels between male and female participants. As shown in **Figure 3**, male and female participants displayed similar levels of total antibody and IgG against the RBD of SARS-CoV-2 S protein and neutralizing antibody at three time points before and after the third dose, suggesting that antibody response induced by the third dose of homologous BBIBP-CoV vaccine was not affected by the sex.

### 3.4 Adverse reactions and events

The injection site and systemic adverse reactions occurring within 7 days after the third dose are shown in **Table 2**. Adverse reactions were reported by 23.71% (23/97) of the total participants. The most common injection site adverse reaction was pain, which was reported by 8 (8.25%) participants; induration at the infection site was reported by one participant (1.03%). The most common systemic adverse reaction was muscle pain, which was reported by 13 (13.40%) participants. In addition to muscle pain, fatigue was reported by 2 (2.06%) participants. All adverse reactions were mild and self-limiting, and no grade 3 adverse reactions were reported.

**Table 2:** Adverse reactions occurring within 7 days after the third dose in participants.

Characteristics	Participants (n=97)
Total reactions after the third dose	
Any, n (%)	23 (23.71)
Grade 3, n (%)	0 (0.00)
Injection site adverse reactions	
Pain, n (%)	8 (8.25)
Induration, n (%)	1 (1.03)
Swelling, n (%)	1 (1.03)
Systemic adverse reactions	
Muscle pain, n (%)	13 (13.40)
Fatigue, n (%)	2 (2.06)
Nausea, n (%)	1 (1.03)

### 3.5 Relationship between total antibody, IgG, IgM, and neutralizing antibody

**Figure 4:**
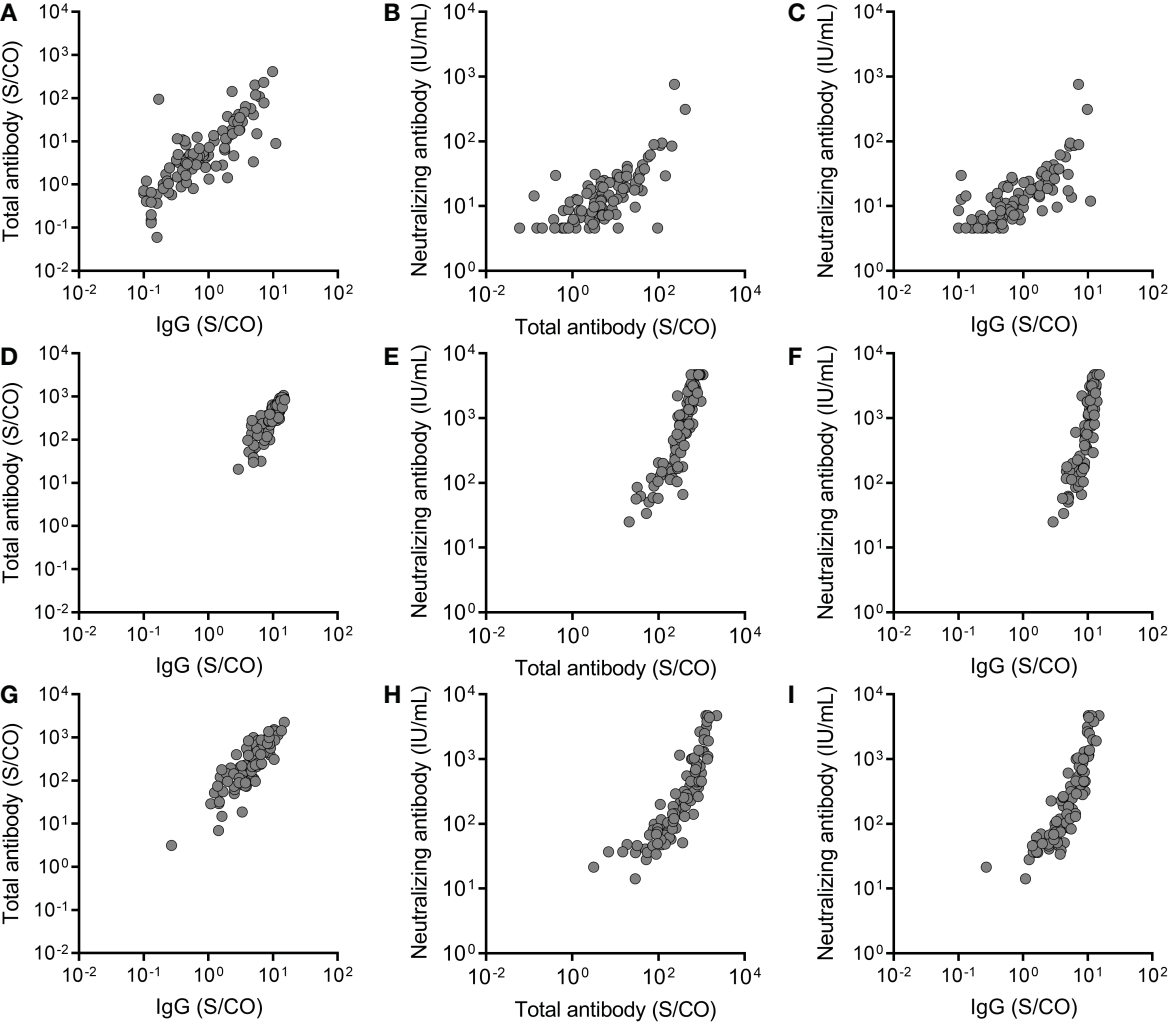
The relationship between total antibody and IgG antibody against the receptor binding domain (RBD) of the SARS-CoV-2 spike (S) protein and neutralizing antibody at three time points, before the third dose **(A-C)**, at month 1 after the third dose **(D-F)** and at month 6 after the third dose **(G-I)**. Spearman rank-correlation analysis was used to analyze the relationship between total antibody and IgG antibody against the RBD of the SARS-CoV-2 S protein and neutralizing antibody against SARS-CoV-2. A significant correlation was observed between total antibody and IgG at three time points (Before the third dose: P <0.0001, r= 0.8130; Month 1 after the third dose: P <0.0001, r= 0.8613; Month 6 after the third dose: P <0.0001, r= 0.8720). Significant correlations were observed between total antibody and IgG and neutralizing antibody before the third dose (for total antibody: P <0.0001, r = 0.7077; for IgG: P < 0.0001, r = 0.7657), at month 1 after the third dose (for total antibody: P <0.0001, r = 0.9243; for IgG: P < 0.0001, r = 0.8919), and at month 6 after the third dose (for total antibody: P <0.0001, r = 0.9339; for IgG: P < 0.0001, r = 0.9255).

The relationship between total antibody, IgG, and IgM against the RBD of SARS-CoV-2 S protein and neutralizing antibody was analyzed. A significant correlation was observed between total antibody and IgG against the RBD of the SARS-CoV-2 S protein at three time points (Before the third dose: P <0.0001, r= 0.8130; Month 1 after the third dose: P <0.0001, r= 0.8613; Month 6 after the third dose: P <0.0001, r= 0.8720) (**Figures 4A, D, G**). Meanwhile, significant correlations between total antibody and IgG against the RBD of the SARS-CoV-2 S protein and neutralizing antibody were observed before the third dose (for total antibody: P <0.0001, r = 0.7077; for IgG: P < 0.0001, r = 0.7657) (**Figures 4B, C**), at month 1 after the third dose (for total antibody: P <0.0001, r = 0.9243; for IgG: P < 0.0001, r = 0.8919) (**Figures 4E, F**), and at month 6 after the third dose (for total antibody: P <0.0001, r = 0.9339; for IgG: P < 0.0001, r = 0.9255) (**Figures 4H, I**). These results suggested that IgG was the main component of total antibody and play an important role in recalling to the BBIBP-CoV vaccine.

## 4 Discussion

Although heterologous booster vaccination induced in more robust immune responses than homologous booster vaccination (24, 25), many people already had their third dose of homologous Covid-19 vaccine. Many people received a third dose of homologous BBIBP-CorV inactivated vaccine in China and other countries. A lot of efforts have been made to investigate the immune response after the homologous BBIBP-CorV booster vaccination (10, 16–19). Given that the neutralizing antibody levels were highly predictive of the host immune protection against infection and correlated with Covid-19 vaccine efficacy (26, 27), the neutralizing antibody responses induced by the homologous BBIBP-CorV booster vaccination were investigated. The neutralizing antibody titer increased rapidly within 4 weeks after the third dose (16). The geometric meant titre of neutralizing antibody against the SARS-CoV-2 ranged from 127-224.4 on 28 days after the third dose (10). In this study, the seropositive rate for neutralizing antibody was 100% at month 1 after the third dose, consistent with the previous study (10). A homologous BBIBP-CorV booster vaccination has been proven to improve neutralization against Omicron variant and provide better protection against the variants of concern (VOC) of SARS-CoV-2 (17–19). Meanwhile, the antibody response and immune persistence induced by the third dose of the BBIBP-CorV inactivated vaccine was not affected by sex, similar to previous studies in which inactivated vaccine elicit similar antibody response regardless of sex (28–30). Previous studies showed that age affected the magnitude of inactivated vaccine-induced antibody response in vaccinees, including the healthcare workers (29–31). However, other studies showed that there was no significant difference in antibody levels in participants of different ages and age was not a factor affecting antibody response induced by inactivated vaccine (28, 32, 33). In this study, the antibody response induced by the BBIBP-CorV inactivated vaccine was not affected by age, similar to the observation in previous studies (28, 32, 33). Robust correlations between the neutralizing antibody and total antibody and IgG against the RBD of SARS-CoV-2 were observed in this study, which is in concordance with previous studies (34, 35), suggesting that IgG and total antibody against the RBD of SARS-CoV-2 were highly predictive for the neutralizing antibody.

It was generally accepted that circulating IgM response was classically transient and IgM was thought to participate in the initial, acute response to viral infections (36), and IgG played an important role in the long-term humoral immunity and immunological memory. IgM response after the third dose was rare in this study, similar to that observed in the previous study in which the seropositive rate of IgM was 9.38% after the third dose of homologous inactivated CoronaVac vaccine (37).

Many studies on the immune persistence after the third dose of Covid-19 vaccine have been conducted (16, 38–41). Previous study showed that the humoral response decay rates after the third dose of Covid-19 vaccine varied among vaccines, but the BBIBP-CorV inactivated vaccine was not included in the study (40). Immunity wanning occurred 10 weeks after the third dose of mRNA Covid-19 vaccine (41). The vaccinees received three doses of the protein subunit vaccine ZF2001 were 100% seropositive against the SARS-CoV-2 prototype isolate at 4 to 7 months after the third dose (42). The seropositive rate of neutralizing antibody was 80.49% at 6 months after the third-dose of homologous CoronaVac inactivated vaccine and neutralizing antibody concentration was 115.23 IU/mL at 9 months after the third dose (38, 39). The neutralizing antibody levels at month 6 after the third dose of homologous BBIBP-CorV inactivated vaccine decreased to 167.83 IU/mL in this study and the positive rate of neutralizing antibody was still 100%. It was important to note that the neutralizing antibody was detected by chemiluminescence microparticle immunoassay in this study and previous studies concerning homologous CoronaVac inactivated vaccine (38, 39) rather than live virus or pseudovirus neutralization test. Due to waning immunity after the booster dose of Covid-19 vaccine, several countries, including the United States, the United Kingdom, and Israel, have begun giving a fourth dose of Covid-19 vaccine to at-risk persons. A real-world study concerning mRNA Covid-19 vaccine has shown that the fourth dose was more effective than the third dose in preventing SARS-CoV-2 infection and severe disease (43). These findings would provide important insights into the development of future vaccination strategies.

A two-dose regimen of the BBIBP-CorV inactivated vaccine conferred 78.1% protection against symptomatic Covid-19 (4), lower than that of the mRNA Covid-19 vaccine (44). While, the BBIBP-CorV inactivated vaccine was one of the safest Covid-19 vaccines currently available in the market. Previous studies have shown that the BBIBP-CorV inactivated vaccine was safe in participants aged ≥3 years, including young people, adults, and elderly, and in participants with comorbidities (4, 10–12). Most of adverse reactions were mild or moderate (4, 10–12). All adverse reactions were mild in this study, similar to observation in previous studies (4, 10, 11, 16, 17).

All participants enrolled in this study had been frequently tested for real-time PCR to detect SARS-CoV-2 infection and had a negative test for SARS-CoV-2 real-time PCR. There were more than 5.2 million people in the study area. Delta resulted in more than 200 laboratory-confirmed cases between August 2021 and September 2021 and Omicron (BA.2) resulted in dozens of laboratory-confirmed cases between March 2022 and April 2022. These suggested effective intervention and control of the Covid-19 pandemic in the study area.

This study does have some limitations. First, the neutralizing antibody titers against the prototype and variants of SARS-CoV-2 were not investigated in this study. Second, this study included the relatively small sample size and most of study participants were female. Third, this study mainly focused on antibody response. However, cellular immunity response induced by the third-dose of homologous BBIBP-CorV inactivated vaccine was not analyzed in this study.

In conclusion, this study showed that a third dose of homologous BBIBP-CorV inactivated vaccine dramatically increase levels of antibody against SARS-CoV-2 without serious adverse events. The neutralizing antibody induced by the third dose of BBIBP-CorV inactivated vaccine could persist for at least 6 months.

## Data availability statement

The raw data supporting the conclusions of this article will be made available by the authors, without undue reservation.

## Ethics statement

The studies involving human participants were reviewed and approved by The Medical Ethics Committee of the Women and Children’s Hospital, School of Medicine, Xiamen University. The patients/participants provided their written informed consent to participate in this study.

## Author contributions

Conceived the study and designed the experiments: H-MY, and Y-LZ. Samples collection: L-RL, MZ, and X-JL. Performed experiments: MZ, and X-JL. Analyzed the data: G-PW. Wrote the first draft of the manuscript: G-PW. All authors contributed to the article and approved the submitted version.
